# Variation in central venous oxygen saturation to assess volume responsiveness in hemodynamically unstable patients under mechanical ventilation: a prospective cohort study

**DOI:** 10.1186/s13054-021-03683-6

**Published:** 2021-07-13

**Authors:** Mohamed Hassene Khalil, Adel Sekma, Wafa Zhani, Asma Zorgati, Houda Ben Soltane, Semir Nouira, the GREAT Network

**Affiliations:** 1 Emergency Department, Fattouma Bourguiba University Hospital, 5000 Monastir, Tunisia; 2 Emergency Department, Sahloul University Hospital, 4011 Sousse, Tunisia; 3 Research Laboratory LR12SP18, University of Monastir, 5019 Monastir, Tunisia; 4 Emergency Department, Farhat Hached University Hospital, 4031 Sousse, Tunisia

*Trial registration*: NCT NCT02142985. Registered 05 May 2014.

Intravenous fluid administration is a cornerstone of hemodynamic resuscitation. Its goal is to restore effective circulating blood volume and correct tissue perfusion as quickly as possible to avoid multi-organ failure [1]. In extreme clinical situations, the diagnosis of hypovolemia is easy; the problem arises when hypovolemia is latent or when associated with a patent left ventricular dysfunction. It is often the case with critically ill patients, for whom the diagnosis of hypovolemia is rarely possible without the use of accurate hemodynamic indicators [2, 3]. The use of central venous oxygen saturation (ScvO_2_) measurement has been proposed to guide fluid therapy [4]. The rationale behind assessing fluid responsiveness by ScvO_2_ is to identify patients on the ascending portion of the Frank-Starling curve who would likely to be fluid-responsive [5]. We studied the ability of SCVO_2_ variation (ΔScvO_2_) to define fluid responsiveness in critically ill emergency department (ED) patients needing volume expansion (VE). Here, we present a comprehensive summary of 88 adult patients under mechanical ventilation who required VE. VE consisted of 500 ml normal saline infused within 10 min. Cardiac output (CO) was measured by thermodilution method before and after VE. Fluid responsiveness was defined as increase in CO ≥ 15% after VE, while fluid non-responsiveness was defined as no increase or increase in CO < 15%. Hemodynamic assessment and blood gases measurements were performed at baseline and immediately after the end of VE. It included heart rate, systolic blood pressure (SBP), diastolic blood pressure (DBP), central venous pressure (CVP), ScvO_2_, CO, cardiac index (CI), oxygen delivery (DO_2_), oxygen consumption (VO_2_) and blood lactate. The most common underlying clinical condition was septic shock. All patients received catecholamine and 46 patients (52.3%) died during hospitalization. Overall, 61 patients (69.3%) responded to VE. Before VE, ScvO_2_ did not differ between responders and non-responders. Patients’ characteristics and hemodynamic variables before VE are summarized in Table1. The increase in SBP, DBP and CI after VE was significantly higher in responders compared to non-responders. CI increased significantly from 2.62 ± 0.75 to 3.47 ± 0.8 L/min/m^2^ (*p* < 0.001) and ScvO_2_ from 70.5 ± 6.8% to 75.2 ± 6.6% (*p* < 0.001) in responders. CI and ScvO_2_ did not change significantly in non-responders after VE. Figure 1 shows the relative changes from baseline of hemodynamic variables after VE. Relative changes of ScvO_2_ were 7 ± 8.4% in responders and − 1.4 ± 9.6% in non-responders. The difference was statistically significant between the two groups (*p* < 0.001). ΔScvO_2_ was positively and significantly correlated with CO variation after VE (*r* = 0.46, *p* < 0.001). When analyzed using multivariate logistic regression, ΔScvO_2_ was the only factor associated with fluid responsiveness [OR: 1.44 (95% CI: 1.15–1.79)]. Diagnostic performance of ΔScvO_2_ and ΔCVP after VE showed areas under ROC curves of 0.84 (95% CI; 0.72–0.96) and 0.56 (95% CI; 0.43–0.68), respectively. The AUC of ΔScvO_2_ was significantly greater than that of ΔCVP (*z* statistic = 3.033, *p* = 0.0024). The best cut-off value found was 4%, allowing discrimination between responders and non-responders with a sensitivity of 78.7%, a specificity of 81.5% and a percentage of correct classification of 61.1%. It is important to mention that our results would work when ScvO_2_ is low and when there is no significant change in VO_2_ and haemoglobin. However, changes depend on the amount of fluid administered. Also, ScvO_2_ may not change if VO_2_ is dependent on DO_2_. We concluded that in patients with acute circulatory failure, ΔScvO_2_ has an adequate correlation with ΔCI and could be very helpful alternative tool when CO measurement or surrogates aren’t possible or not applicable. Further studies with larger populations are required to confirm these results on the role of ScvO_2_ monitoring in assessing fluid responsiveness instead of a cardiac output measurement during a fluid challenge.

**Table 1:** Baseline characteristics and hemodynamic variables before volume expansion in the responders and the non-responders groups

	Overall population *N* = 88	Responders *n* = 61	Non-responders *n* = 27	*p*
Age (SD)	70 ± 13	71 ± 14	67 ± 11	0.14
Male (%)	65 (73.9)	46(75.4)	19 (70.4)	0.61
Underlying diseases n (%)				
Heart failure	19 (21.6)	12 (25)	5(19.2)	0.77
Arterial hypertension	47 (53.4)	24 (50)	16 (61.5)	0.46
Diabetes	72 (81.8)	42 (87.5)	18 (69.2)	0.06
Coronary artery disease	27 (30.7)	13 (27.1)	8 (30.8)	0.79
SOFA score mean (SD)	15 ± 3	15 ± 3	15 ± 3	0.75
SAPS II score mean (SD)	75 ± 25	75 ± 24	76 ± 25	0.85
Type of shock				
Septic (%)	34 (38.6)	26 (42.6)	8 (29.6)	0.34
Hypovolemic (%)	11 (12.5)	7 (11.5)	4(14.8)	0.73
Cardiogenic (%)	10 (11.4)	6 (9.8)	4 (14.8)	0.48
Combined (%)	33 (37.5)	22 (36.1)	11 (40.7)	0.81
Death (%)	46 (52.3)	31(50.8)	15 (55.6)	0.82
Heart rate (bpm)	110 ± 19	109 ± 18	111 ± 22	0.61
Systolic arterial pressure mmHg, mean (SD)	91.3 ± 10	91.3 ± 8	91.3 ± 12	0.99
Diastolic arterial pressure mmHg, mean(SD)	54.2 ± 9	51.2 ± 8	61.1 ± 8	< 0.001
Central venous pressure cmH_2_O mean (SD)	7.95 ± 2.38	6.8 ± 1.48	10.6 ± 1.88	< 0.001
Cardiac output (L/min)	4.88 ± 1.52	4.97 ± 1.42	4.67 ± 1.75	0.39
Cardiac index (L/min/m^2^) mean (SD)	2.57 ± 0.8	2.6 ± 0.74	2.46 ± 0.92	0.39
Oxygen delivery (ml/kg/min) mean (SD)	446.2 ± 150	497.5 ± 131	330.5 ± 126	< 0.001
Oxygen consumption (ml/kg/min) mean (SD)	120.5 ± 51	137.3 ± 54	95.5 ± 44	0.001
Lactate (mmol/l) mean(SD)	3.6 ± 2.58	3.5 ± 2.31	3.9 ± 3.05	0.53
ScvO_2_ (%)	70.8 ± 7	70.5 ± 7	71.5 ± 8	0.51

SOFA, Sequential Organ Failure Assessment, SAPS, Simplified acute physiology Score, ScvO_2_, central venous oxygen saturation

**Fig. 1:**
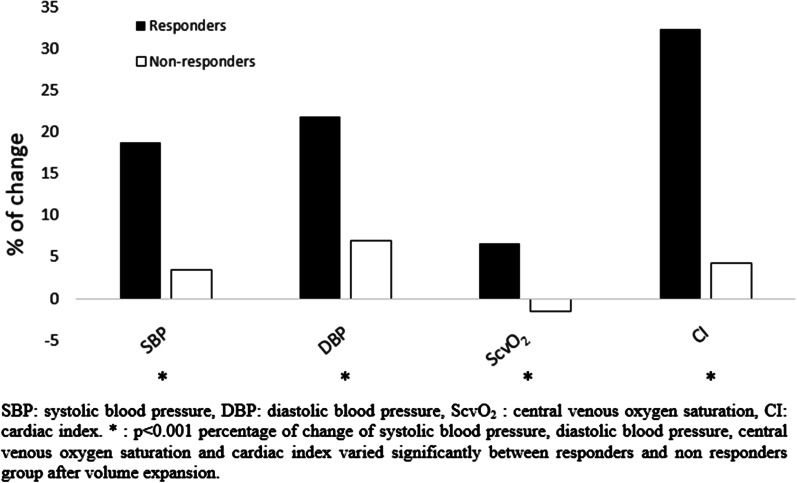
Relative changes from baseline of hemodynamic variables after volume expansion

## Data Availability

The data are fully available, please contact the corresponding author.

## Abbreviations

ScvO_2_: Central venous oxygen saturation
∆ScvO_2_: ScvO_2_ variation
CO: Cardiac output
CI: Cardiac index
∆CI: Cardiac index variation
ED: Emergency department
VE: Volume expansion
VO_2_: Oxygen consumption
DO_2_: Oxygen delivery
SBP: Systolic blood pressure
DBP: Diastolic blood pressure
CI: Cardiac index
CVP: Central venous pressure
ΔCVP: CVP variation
